# Update/Refinement of Targeted Muscle Reinnervation Indication: A Scoping Review of Applications for Non-Amputees

**DOI:** 10.3390/jcm13206107

**Published:** 2024-10-14

**Authors:** Jonathan Cornacchini, Haïzam Oubari, Vlad Tereshenko, Maria Bejar-Chapa, Yanis Berkane, Anna Scarabosio, Alexandre G. Lellouch, Olivier Camuzard, Kyle R. Eberlin, Elise Lupon

**Affiliations:** 1 Department of Plastic and Reconstructive Surgery, Pasteur University Hospital, 06000 Nice, France; jcornacchini@mgh.harvard.edu (J.C.); camuzard.olivier@hotmail.fr (O.C.); elise.lupon@gmail.com (E.L.); 2 Vascularized Composite Allograft Laboratory, Massachusetts General Hospital and Harvard Medical School, Boston, MA 02114, USA; houbari@mgh.harvard.edu (H.O.); yberkane@mgh.harvard.edu (Y.B.); alellouch@mgb.org (A.G.L.); 3 Department of Plastic, Reconstructive and Aesthetic Surgery, Grenobles University Hospital Center, 38700 Grenobles, France; 4 Department of Plastic and Reconstructive Surgery, Massachusetts General Hospital and Harvard Medical School, Boston, MA 02114, USA; vtereshenko@mgh.harvard.edu (V.T.); mbejarchapa@mgh.harvard.edu (M.B.-C.); keberlin@mgh.harvard.edu (K.R.E.); 5 Department of Plastic, Reconstructive and Aesthetic Surgery, Rennes University Hospital Center, 35033 Rennes, France; 6 Department of Plastic and Reconstructive Surgery, Ospedale Santa Maria della Misericordia, 33100 Udine, Italy

**Keywords:** TMR, non-amputee, neuroma, complex regional pain syndrome, post-mastectomy pain syndrome, neuropathic pain

## Abstract

**Background:** Targeted muscle reinnervation (TMR) was originally developed to enhance prosthetic control in amputees. However, it has also serendipitously demonstrated benefits in reducing phantom pain and neuromas. As a result, it has emerged as a secondary treatment for chronic neuromas in amputees and holds promise for managing neuropathic pain in non-amputee patients, particularly those with neuromas. This review synthesizes the current literature on TMR indications for non-amputee patients, highlighting its potential to address chronic peripheral nerve pain and neuromas beyond its original application in amputation. **Methods:** A thorough search of the PubMed and Cochrane databases up to January 2024 was conducted following the PRISMA guidelines. Inclusion criteria comprised case series, cohort studies, and randomized controlled trials reporting TMR outcomes in non-amputees. **Results:** Of 263 articles initially identified, 8 met the inclusion criteria after screening and full-text assessment. The articles were all case series with varied sample sizes and mainly focused on neuroma treatment (n = 6) and neuropathic pain management (n = 2) for both upper and lower extremities. Clinical studies included TMR efficacy for sural nerve neuromas in the lower extremities and hand neuromas, showing pain relief and improved function. Key findings were encouraging, showing successful pain relief, patient satisfaction, and psychosocial improvement, with only rare occurrences of complications such as motor deficits. **Conclusions:** In non-amputee patients, TMR appears to be a promising option for the surgical management of neuropathic pain, demonstrating favorable patient satisfaction and psychosocial outcomes along with low morbidity rates. Although functional improvements in gait recovery and range of motion are encouraging, further research will be important to confirm and expand upon these findings.

## 1. Introduction

Targeted muscle reinnervation (TMR) is a technique that was first described by Dumanian et al. [1,2] in 2004 to improve the control and dexterity of myoelectric prostheses in amputee patients. The technique involves the coaptation of major peripheral nerves to a recipient motor branch that supplies an expendable muscle target [3]. In addition, TMR has been described as an effective technique to reduce phantom pain and neuroma formation [4,5] in amputee patients, especially in cases where traditional neuroma excision and nerve reconstruction are either not feasible, have proven unsuccessful, or have failed. The presence of a symptomatic neuroma, which can develop after a nerve injury [6] due to compression, trauma, or surgery, may be characterized by tenderness in an area with an incision scar [7]. Common clinical anatomic locations include superficial radial neuroma, superficial peroneal, saphenous, and or sural neuromas following foot and ankle surgery, and saphenous neuroma in the thigh after knee arthroscopy or total knee arthroplasty [8]. Since TMR has been initially implemented in amputees, it is often perceived as being specific to this patient population. However, any patient with chronic peripheral nerve pain (regardless of amputation) could potentially benefit from TMR, especially when considering the treatment of neuromas. The application of this technique in non-amputee patients has yet to be a comprehensive synthesis of the available studies. This article aims to identify and synthesize the currently available literature on TMR indications for non-amputee patients.

## 2. Materials and Methods

### 2.1. Study Design

This scoping review was conducted in accordance with the Preferred Reporting Items for Systematic Reviews and Meta-Analyses (PRISMA) guidelines [9]. We performed a comprehensive search of the electronic databases MEDLINE (PubMed), Embase, and Cochrane Library from their inception up to January 2024. The following search terms were used: (“Targeted Muscle Reinnervation” OR “TMR”) AND (“Indication” OR “Non-amputee”). Additionally, we conducted manual searches of reference lists from identified articles and relevant review papers to ensure thorough coverage. The search was restricted to articles published in English. The initially selected articles were screened by title and abstract to remove duplicates and non-English studies. Subsequently, reviews, animal studies, and irrelevant papers were excluded. Full-text articles were then reviewed, and case reports as well as anatomical studies were eliminated.

### 2.2. Eligibility Criteria 

Studies were included based on the following criteria:

- **Types of Studies**: All studies (anatomical, case reports, case series, cohort studies, and randomized controlled trials) reporting outcomes of TMR in non-amputee patients for indications other than amputation were initially included. Review articles, conference abstracts, case reports, and animal studies were excluded.
- **Participants**: Studies involving both pediatric and adult patients undergoing TMR were included.
- **Outcome Measures**: Studies reporting relevant outcomes such as pain relief, absence of motor deficits, complications, patient satisfaction, and psychosocial outcomes were included.

### 2.3. Study Selection 

Two independent reviewers (J.C. and H.O.) screened the titles and abstracts of identified articles to assess their eligibility based on the predetermined inclusion and exclusion criteria. Full-text articles of potentially eligible studies were retrieved and reviewed in detail. Any disagreements were addressed through discussion and, if necessary, by consulting a third reviewer.

### 2.4. Data Extraction 

Data from eligible studies were extracted using a standardized data extraction form. The following information was collected: study characteristics (author, year, and country), study design, participant demographics, sample size, etiology, indication, adjuvant techniques, outcome measures assessed, follow-up duration, and reported results. Two reviewers performed data extraction independently, and any discrepancies were resolved through consensus.

### 2.5. Quality Assessment 

Discrepancies in the quality assessments were resolved through discussion and, if necessary, a third reviewer was consulted to reach consensus. 

### 2.6. Data Synthesis and Analysis 

Given the heterogeneity in study designs and outcome measures, a meta-analysis was not favored. Instead, a narrative synthesis of the included studies was conducted. Data were organized and summarized in a tabular format, highlighting the characteristics of the included studies, key findings, and outcomes of interest. Where appropriate, subgroup sensitivity analyses were performed to explore sources of heterogeneity and assess the robustness of the findings.

## 3. Results

### 3.1. Study Selection 

**Figure 1:**
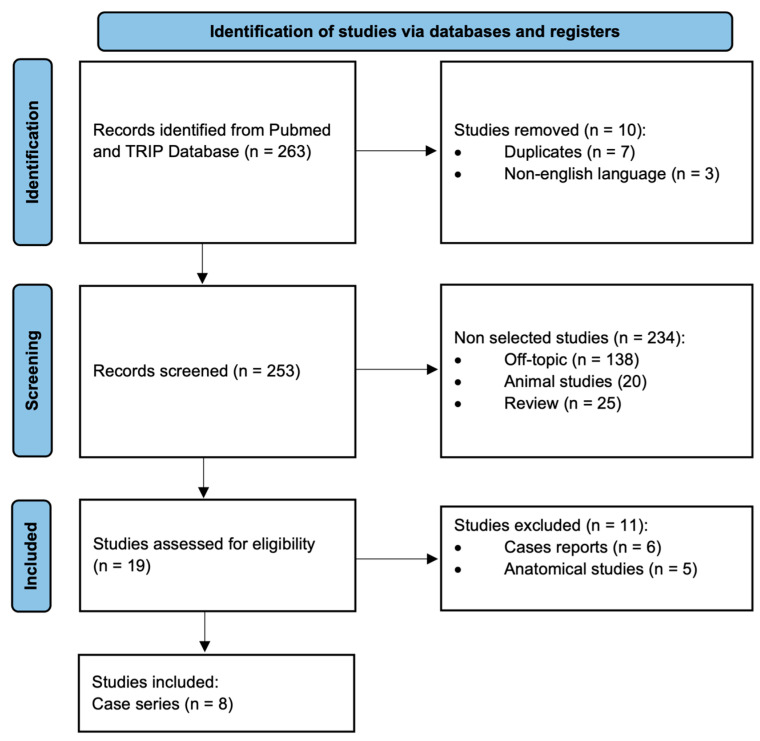
Flowchart.

An initial search of PubMed and TRIP Database sources identified 263 articles. After removing duplicates, 253 unique articles were available for screening. Titles and abstracts were reviewed, leading to the retrieval of 19 articles for full-text assessment. Of these, 11 articles were excluded: 5 were anatomical studies and 6 were case reports. Finally, eight studies met the predefined inclusion and exclusion criteria and were included in the review (the flowchart is available in Figure 1).

### 3.2. Study Characteristics

All the included studies were case series predominantly conducted in the United States (n = 7). Sample sizes varied across the studies, ranging from 6 to 15 participants, with a total of 68 participants overall. All studies were relatively recent, with the earliest publication dating back to 2020. 

### 3.3. Indications and Anatomical Location and Techniques 

Among the included articles, four specifically focused on neuroma treatment or prevention [10,11,12,13], whereas only two dealt with neuropathic pain and complex regional pain syndrome (CRPS) [14,15]. Additionally, two publications examined specific conditions such as post-mastectomy pain syndrome and thoracic outlet syndrome [16,17]. Most of the studies investigated conditions in the extremities [11,12,13,14,15] (n = 5) (Table 1).

### 3.4. Upper Extremity 

The first clinical paper on this topic, published in 2020, investigated the restoration of sensation to and the minimization of pain levels in the hand before and after TMR using contralateral C7 nerve transfers [14]. This case series focused on patients presenting traumatic brachial plexus injuries with C5 rupture and avulsion of C6, C7, and C8. The traumatic events primarily resulted from motor vehicle and railroad accidents. Eleven patients with plexopathy underwent contralateral transfer of C7 to the median nerve with a sural graft. Concurrently, a C5 or C6 free graft was performed in the posterior cord, along with nerve transfers from the spinal accessory nerve to the suprascapular nerve and intercostal nerves T4, T5, and T6 to the musculocutaneous nerve. After a mean follow-up of 7 years, 73.6% of patients showed improvements in their Brief Pain Inventory (BPI) scores, and sensory function improved in ten out of eleven patients. However, only one patient achieved motor recovery. A study conducted in 2022 established the effectiveness of TMR on the distal branches of the extensor carpi radialis brevis (ECRB) to treat neuromas of the superficial branch of the radial nerve (SRN) while preserving function in six patients [12]. The causes of this condition can vary, including trauma, intravenous cannulation, De Quervain release, Kirschner wire fixation of distal radius fractures, and carpal surgery [18], leading to neuropathic pain. With follow-up periods ranging from 5 to 20 months, patients experienced a reduction in Visual Analog Scale (VAS) scores in pain, which improved from a range of 7–10 to 0–4. Additionally, all patients retained full wrist extension strength (5 out of 5). Unfortunately, this article provides minimal information about patient outcomes and follow-up. While the technique is described, the discussion only briefly touches on outcomes and follow-up in the other cases. Another article [13] explored the utilization of TMR for managing wrist neuromas involving the SRN, the palmar cutaneous branch of the median nerve (PBCMN), and the median nerve. The study included eight patients with various underlying conditions and surgical histories, such as De Quervain tenosynovitis release, traumatic wrist laceration, and volar wrist ganglion excision. Coaptation of the anterior interosseus nerve (AIN) to the pronator quadratus (at) was performed. The primary outcome measure was pain relief, which was assessed using the VAS. The average preoperative pain score of 7/10 significantly improved to 2/10 postoperatively in seven patients.

### 3.5. Neck

Bombardelli et al. discussed the TMR of the supraclavicular nerve (SCN) [16] to the motor branch of the omohyoid muscle in 10 patients who manifested neurogenic thoracic outlet syndrome (NTOS). Due to the potential for desiccation and neuroma formation following a suprascapular incision during surgical treatment of this syndrome, the authors performed targeted muscle reinnervation (TMR) concomitantly with surgery in 10 patients to assess the incidence and severity of postoperative pain. After an average follow-up of 8 months, no supraclavicular pain or hypersensitivity was reported by patients. Additionally, there was a gradual improvement in strength and a return of sensation in the arm and hand on the operated side.

### 3.6. Breast

O’Brien et al. reported on the results of TMR for pain treatment following mastectomy in 11 patients [17]. The study involved the transfer of the cutaneous breast intercostal nerves to the serratus anterior, intercostal, or pectoral muscles, with the goal of preventing post-mastectomy pain syndrome. Four patients achieved an average BREAST-Q score of 77.5/100, which is notably higher than the expected score for mastectomy patients, as observed in a study of 1201 women [19]. However, the report did not specify the BREAST-Q scores prior to mastectomy and TMR surgery in these patients.

### 3.7. Abdomen 

Chappell et al. reported on a series of 20 patients who experienced groin pain due to abdominal wall neuromas after abdominal or hernia surgery. Eight of these patients were treated with TMR [10] of the ilioinguinal and/or genitofemoral nerves to a motor nerve of the internal oblique. At the last follow-up visit (ranging from 5.45 to 43.65 months), positive results were observed in seven out of eight patients. These outcomes included being pain-free (n = 3), significant pain improvement (n = 1), improvement with a change in pain quality (n = 1), reduction in pain with cessation of preoperative pain medications (n = 1), and being pain-free after peripheral nerve stimulator placement (n = 1). One patient reported new incisional pain post surgery, but no additional details were provided on the evolution of preoperative pain.

### 3.8. Studies with Multiple Locations 

The first study to assess TMR for complex regional pain syndrome (CRPS) type 2 in limbs was published in 2022 [15]. Among the 13 patients, 7 were non-amputees and exhibited various causes of neuromas, including De Quervain release, wrist trauma reconstruction, triangular fibrocartilage complex (TFCC) trauma, leg trauma, and carpal tunnel release. In non-amputee patients, the follow-up ranged from < 1 month to 17 months. The Numerical Pain Rating Scale showed improvement for the non-amputee patients, and an improvement in function without more precision was mentioned for all patients. Therefore, several other therapies were administered to these patients, such as radial sensory neuroma excision, sural nerve grafting, dorsal cutaneous neuroma excision, SPN neurectomy, sural neurectomy, posterior interosseous nerve decompression, median neuroma excision, LAB excision, and radial nerve decompression. Also, a retrospective series of 15 non-amputee patients was conducted to assess the effectiveness of TMR for treating neuromas in the limbs [11]. This study included patients with symptomatic neuromas treated with TMR from 1 January 2019 to 1 January 2020. Significant improvements were observed in both the frequency and severity of pain. The average follow-up period was 8.1 months, during which no postoperative complications were reported, and numbness in the affected dermatome was well tolerated. Additionally, one patient who developed a neuroma of the intercostal nerve after cholecystectomy underwent TMR of the rectus abdominis muscle.

## 4. Discussion

This scoping review identified and included eight studies, primarily case series conducted in the United States. While most patients showed significant improvements in pain scores and sensory function, motor recovery was less consistently achieved. The findings underscore the potential of TMR as a promising technique for managing neuropathic pain across diverse anatomical regions, although variations in outcomes highlight the need for further research to optimize its efficacy and application.

The primary outcomes assessed in the included studies were pain level and/or pain relief, as well as sensory improvement (n = 8, including BREAST-Q). However, it is important to point out that functional outcomes were not consistently evaluated in these studies (n = 5). Despite variations in measurement methods and reporting, nearly all studies (7 out of 8) reported significant pain relief in every patient, using either the VAS (Visual Analog Scale), the NRS (Numeric Rating Scale), or unspecified 0–10 pain scores. The mean patient follow-up ranged from 5 months to over 6 years. The primary limitations stem from the heterogeneity in study designs, patient populations, and reported outcomes, making it difficult to draw definitive conclusions regarding the efficacy of TMR.

Regarding the donor site, complaints and complications remained minimal, with no motor morbidity, as evidenced by the satisfactory motor function and range of motion when mentioned. Patients’ complaints about the procedure were infrequent; only 1 [15] among the 68 non-amputatee patients reported worsening pain. This case underwent a contralateral root transfer from C7, which is relatively uncommon in TMR procedures and is typically associated with other interventions such as additional nerve transfers, grafts, and functional muscle transfers. Undesired, though anticipated, events when reported were limited to donor nerve impairment such as numbness and muscle denervation [15]. However, complications were briefly mentioned in every study except one [12]. Across these studies, there were only a few cases of transient [14] and permanent [13,16] numbness in the sensory area of the transected nerve. In the Chang et al. study, this numbness was considered preferable to the initial pain and was not bothersome to the patients. 

Interestingly, the use of nerve allografts for ilioinguinal nerve reconstruction was described in the study by Chappell et al. [10]. However, this approach failed to relieve the patients’ pain, ultimately requiring a TMR procedure on the internal oblique nerve to achieve an improvement in pain. Besides allografts, other adjuvant therapies were mentioned in different publications (n = 3), including neurectomy, neuroma excision, neurolysis, and graft neuroplasty [10,14,15,20], which complicates assessments of the specific role of TMR in pain relief. Only two studies reported patient satisfaction and quality of life following TMR procedures [11,17]. Overall, most patients reported high levels of satisfaction with the procedure. Significant improvements in psychological well-being, social functioning, and quality of life were observed postoperatively in the assessed studies. The studies predominantly involved upper- and lower-extremity cases. Interestingly, some studies also focused on other anatomical sites, including the breast [17] (post mastectomy), neck [16] (NTOS), and abdomen [10] (groin pain or post-surgery sequelae).

The different indications for TMR were post-surgical (sural nerve grafts, ganglion or tumor resections, inguinal hernia repairs, mastectomies, hysterectomies, cholecystectomies, mastectomy, hysterectomy, cholecystectomy, thoracic outlet syndrome procedures, knee replacements, bunionectomies, De Quervain tenosynovitis, carpal tunnel releases, etc.) and traumatic injuries such as complex fractures, leading to neuromas or CRPS. TMR appears to be more effective for managing neuromas compared to CRPS, which is often complicated by factors such as pseudoarthrosis and tenosynovitis, resulting in a more complex treatment scenario.

Previous reviews have been mostly focused on amputee cases [3,21,22], and only one in 2021 briefly exposed the use of TMR in the treatment of neuroma in non-amputee patients [23]. Consequently, despite the positive outcomes observed for CRPS, further investigation is warranted to fully explore the potential benefits of TMR for this indication.

### 4.1. Potential TMR Applications to Consider 

#### 4.1.1. Lower Extremity

Anatomical studies suggest that TMR could be a viable treatment for Morton’s neuroma, which is characterized by perineural fibrosis and hyperplasia of the deep transverse metatarsal ligament [24]. Three studies have demonstrated the feasibility of this approach. Two studies [25,26], each involving five adult cadavers, showed that the length of the common digital nerves may be adequate for coaptation with the deep branch of the lateral plantar nerve. Another study [27], also conducted on five cadaveric lower extremities, identified the average number of nerves supplying the flexor digitorum brevis (FDB) and abductor hallucis (AH) (2 and 2.2 nerves, respectively). This study also noted two potential incision points for accessing these nerves. A 2018 study outlined optimal incision placements and motor nerve targets in the leg [28]. It identified the extensor digitorum longus (EDL) as the primary target in the anterior compartment, noting 3.0 motor entry points covering 20 to 80 percent of the leg length, which was attributed to the expandable functional nature of this muscle. The peroneus longus was preferred in the lateral compartment, providing 5.8 motor entry points across 20 to 70 percent of the leg length. In the superficial posterior compartment, the gastrocnemius muscles were targeted, with approximately four motor entry points on each side covering 0 to 40 percent of the leg length. The deep posterior compartment was less utilized in non-amputees.

The clinical application of the gastrocnemius motor branch as a target for TMR has shown promising results in managing sural nerve neuromas. In one case [25], a patient with significant neuropathic pain experienced complete pain relief and resumed normal ambulation within six months. Another case [29] demonstrated the technique’s effectiveness in alleviating persistent pain following Achilles surgery, with significantly reduced pain and no weakness in plantar flexion (Medical Research Council (MRC) grade 5). A case series on saphenous nerve reconstruction was published in 2020 [30], highlighting the use of targeted muscle reinnervation (TMR), particularly on the motor nerve branch to the vastus medialis, as a final solution. Among the 18 patients included, only one was a non-amputee, which is why we decided not to include this case in our analysis.

#### 4.1.2. Upper Extremity

Regarding the use of the PQ as a TMR target, two studies included in this review demonstrated its efficacy. Additionally, the literature supports the PQ as a solution for neuromas of the PCBMN after carpal tunnel release [31] or fracture [20]. The PQ also showed potential as a target for addressing neuromas associated with the dorsal branch of the ulnar nerve (DBUN), as evidenced by a case study involving a patient post ganglion removal surgery [32]. Furthermore, an anatomical study on two cadavers suggested its relevance for neuromas of the distal lateral antebrachial cutaneous nerve, SRN, and PCBMN [31]. An extensive anatomical study on seven upper limbs extensively analyzed the course of the SRN in the forearm and the motor nerve supply to the PQ, BR, ECRL, and ECRB muscles [33]. This study found that the RSN can be transferred to the PQ if the neuroma is located in the distal third of the forearm. Alternatively, the SRN can be directed to the motor branches of the ECRL, or to the BR and ECRB muscles. Another potential application of TMR could be in cases of medial antebrachial cutaneous neuroma, particularly following ulnar nerve decompression, elbow surgery, or brachioplasty, with the flexor carpi ulnaris as the target nerve. A V-shaped incision following the course of the pronator teres, advancing over the medial epicondyle, and extending toward the biceps can be performed.

#### 4.1.3. Head and Neck

Gfrerer et al. detailed a technique for addressing occipital neuromas in cases of refractory migraine pain [34]. Their approach involved decompression, neuroma excision, and grafting with TMR onto the splenius capitis muscle. However, no outcomes have been reported yet, underscoring the need for further research in this area. Regarding the alternative available therapies, a study published in 2023 [35] compared the outcomes of RNPI and TMR in the treatment of neuroma in 36 rats. Following transection of the tibial nerve and its fixation to the dermis to promote neuroma formation, either RNPI or TMR was performed. Pain was assessed through mechanical stimulation at the neuroma site, using the thigh tap and Von Frey tests, which revealed no significant differences in pain scores between the two methods. Consequently, the choice between these techniques may depend more on practical considerations, such as ensuring sufficient nerve length and size for TMR and survival of the muscle graft for RNPI.

## 5. Conclusions

The results of this scoping review suggest that TMR can be an effective approach for pain management in non-amputees. Patient generally reported high levels of satisfaction and positive psychosocial outcomes, with a low morbidity rate. Functional outcomes also indicated promising improvements in gait and range of motion. However, further research is needed to strengthen the evidence in these areas.

## Figures and Tables

**Table 1:** Studies investigated.

Article	Patient (n)	Study Type	Etiology	Indication—Localisation	Nerves Involved	Adjuvant Therapies	Outcomes	Complications	Approximate Mean Follow-Up (Months)
Monsivais et al. 2020 [14]	11	Case series	Traumatisms: MVA, railroad, bicycle	C4-T11 plexopathy, neuropathic pain	C7 contralateral root to median nerve	SN grafting C5-C6 grafting Intercostals 5, 6, and 7→MC C5→SCN, MC	BPI improvement: 73.6% Pain VAS: Postoperative: 1–9 Preoperative: 8–10 MRS: 1–4	Transient hypoesthesia on the donor side, mid-palm, and digits	82.8
Chappell et al. 2021 [10]	8	Case series	Surgery: Inguinal hernia repairs, hysterectomy, cholecystectomy	Abdominal wall neuromas	Ilioinguinal, genitofemoral, iliohypogastric nerve→Internal oblique nerve	Nerve allograft: ilioinguinal and genitofemoral	Pain improved significantly, pain-free, change in pain	None	18.9
Bombardelli et al. 2022 [16]	10	Case series	NTOS: Traumatic, idiopathic, orthopedic surgery, mass effect	SCN neuromas	SCN→OM	N/A	Progressive improvement in strength, recovery of sensation in the arm and hand	None	8
O’Brien et al. 2020 [17]	11	Case series	Surgery: Mastectomy	PMPS	IN→Serratus anterior, adjacent intercostal muscle, pectoralis minor and major	N/A	BREAST-Q survey mean score: 77.5/100	None	8.5
Chang et al. 2021 [11]	15	Case series	Hand, orthopedic or plastic surgery, traumatisms, laparoscopic cholecystectomy	UE, LE, and trunk neuromas	RSN→ECRB DCBUN→FCU 3CDN→QP SPN→EDL SPN→Peroneous brevis SPN→Tibial ant DPN→EHL SN→Medial gastrocnemius SN→Peroneous brevis SaN→Sartorius SaN→Medial gastrocnemius IN→Rectus abdominis	N/A	Mean pain VAS: Postoperative: 7.9 Preoperative: 4.3 Overall physical function: Postoperative: 3.7 Preoperative: 5.8 Overall quality of life: Postoperative: 4.9 Preoperative: 7.0 Motor function: 5/5 strength	Numbness in the corresponding dermatome	8.1
Cox et al. 2023 [12]	6	Case series	Wrist and hand surgery, traumatism	RSN neuromas	RSN→ECRB	N/A	Pain VAS: Postoperative: 7–10 Preoperative: 0–4 Motor function: 5/5 wrist extension	N/A	12.5
Shin et al. 2022 [15]	7	Case series	Wrist and hand surgery	CRPS II	SPN; SaN; SN; RSN; median; LAB cut	Neuroplasty with graft, neuroma excision (RSN, median, LAB), neurectomy (SPN, SM), nerve decompression (posterior interosseux, RSN)	Mean NRS improvement: 2.75 ± 3.41/10 (worsening in one patient), improvement in function	General pain, shooting pain, burning pain, numbness, Hyper- and cold sensitivity Tingling	8.5
Muneer et al. 2024 [13]	8	Case series	Wrist surgery, traumatisms	Wrist neuromas	SRN, PCBMN, Median→AIN (PQ)	N/A	Mean Pain VAS: Preoperative: 7/10 Postoperative: 2/10 Physical health and quality of life improved significantly	Numbness along the corresponding dermatome (7)	13

MVA: Motor Vehicle Accident; VAS: Visual Analog Scale; BPI: Brief Pain Inventory; MRS: Motor Recovery Score; NTOS: Neurogenic Thoracic Outlet Syndrome; SCN: Suprascapular Nerve; PMPS: Post-Mastectomy Syndrome; UEs: Upper Extremities; LEs: Lower Extremities; SRN: Sensitive Radial Nerve; CRPS: Complexe Regional Pain Syndrome; LAB: Lateral Antebrachial Nerve; OM: Omohyoidis Muscle Nerve; ECRB: Extensor Carpi Radialis Brevis; EDL: Extensor Digitorum Longus; EHL: Extensor Hallucis Longus; PCBMN: Palmar Cutaneous Branch of the Median Nerve; AIN: Anterior Interosseus Nerve; PQ: Pronator Quadratus; DBUN: Dorsal Cutaneous Branch of Ulnar Nerve; FCU: Flexor Carpi Ulnaris; 3CDN: 3rd Common Webspace Digital Nerve; SPN: Superficial Peroneal Nerve; DPN: Deep Peroneal Nerve; SN: Sural Nerve; SaN: Saphenous Nerve; MC: Musculocutaneous; NRS: Numerical Pain Rating Scale; IN: Intercostal.

## Data Availability

No new data have been created.
